# Supplementary material for: OpenMM 7: Rapid development of high performance algorithms for molecular dynamics
Source: PLoS Comput Biol. 2017 Jul 26;13(7):e1005659. doi: 10.1371/journal.pcbi.1005659 (PMC5549999; doi:10.1371/journal.pcbi.1005659)
Supplement: S1 Source Code — (ZIP) [file pcbi.1005659.s002.zip › OpenMM-7.0.1-Source/docs/api-python/generated/simtk.openmm.openmm.CompoundIntegrator.html]

CompoundIntegrator — OpenMM 7.0.1.dev-5e86c4f documentation


# CompoundIntegrator¶

*class* `simtk.openmm.openmm.``CompoundIntegrator`(*\*args*)¶
:   This class allows you to use multiple integration algorithms within a single simulation, switching back and forth between them. To use it, create whatever other Integrators you need, then add all of them to a CustomIntegrator:

    ```
    CompoundIntegrator compoundIntegrator;
    compoundIntegrator.addIntegrator(new VerletIntegrator(0.001));
    compoundIntegrator.addIntegrator(new LangevinIntegrator(300.0, 1.0, 0.001));
    ```

    Next create a Context, specifying the CompoundIntegrator as the Integrator to use for the Context:

    ```
    Context context(system, compoundIntegrator);
    ```

    Finally, call setCurrentIntegrator() to set which Integrator is active. That one will be used for all calls to step() until the next time you change it.

    ```
    compoundIntegrator.setCurrentIntegrator(0);
    compoundIntegrator.step(1000); // Take 1000 steps of Verlet dynamics
    compoundIntegrator.setCurrentIntegrator(1);
    compoundIntegrator.step(1000); // Take 1000 steps of Langevin dynamics
    ```

    When switching between integrators, it is important to make sure they are compatible with each other, and that they will interpret the positions and velocities in the same way. Remember that leapfrog style integrators assume the positions and velocities are offset from each other by half a time step. When switching between a leapfrog and non-leapfrog integrator, you must first adjust the velocities to avoid introducing error. This is also true when switching between two leapfrog integrators that use different step sizes, since they will interpret the velocities as corresponding to different times.

    `__init__`(*self*) → CompoundIntegrator¶
    :   \_\_init\_\_(self, other) -> CompoundIntegrator

        Create a CompoundIntegrator.

    Methods

    |  |  |
    | --- | --- |
    | `__init__`((self) -> CompoundIntegrator) | \_\_init\_\_(self, other) -> CompoundIntegrator |
    | `addIntegrator`((self, integrator) -> int) | Add an Integrator to this CompoundIntegrator. |
    | `getConstraintTolerance`((self) -> double) | Get the distance tolerance within which constraints are maintained, as a fraction of the constrained distance. |
    | `getCurrentIntegrator`((self) -> int) | Get the index of the current Integrator. |
    | `getIntegrator`((self, index) -> Integrator) | getIntegrator(self, index) -> Integrator |
    | `getNumIntegrators`((self) -> int) | Get the number of Integrators that have been added to this CompoundIntegrator. |
    | `getStepSize`((self) -> double) | Get the size of each time step, in picoseconds. |
    | `setConstraintTolerance`(self, tol) | Set the distance tolerance within which constraints are maintained, as a fraction of the constrained distance. |
    | `setCurrentIntegrator`(self, index) | Set the current Integrator. |
    | `setStepSize`(self, size) | Set the size of each time step, in picoseconds. |
    | `step`(self, steps) | Advance a simulation through time by taking a series of time steps. |

    `getNumIntegrators`(*self*) → int¶
    :   Get the number of Integrators that have been added to this CompoundIntegrator.

    `addIntegrator`(*self*, *integrator*) → int¶
    :   Add an Integrator to this CompoundIntegrator. The Integrator object should have been created on the heap with the “new” operator. The CompoundIntegrator takes over ownership of it, and deletes it when the CompoundIntegrator itself is deleted. All Integrators must be added before the Context is created.

        |  |  |
        | --- | --- |
        | Parameters: | **integrator** (*Integrator \**) – the Integrator to add |
        | Returns: | the index of the Integrator that was added |
        | Return type: | int |

    `getIntegrator`(*self*, *index*) → Integrator¶
    :   getIntegrator(self, index) -> Integrator

        Get a const reference to one of the Integrators that have been added to this CompoundIntegrator.

        |  |  |
        | --- | --- |
        | Parameters: | **index** (*int*) – the index of the Integrator to get |

    `getCurrentIntegrator`(*self*) → int¶
    :   Get the index of the current Integrator.

    `setCurrentIntegrator`(*self*, *index*)¶
    :   Set the current Integrator.

        |  |  |
        | --- | --- |
        | Parameters: | **index** (*int*) – the index of the Integrator to use |

    `getStepSize`(*self*) → double¶
    :   Get the size of each time step, in picoseconds. This method calls getStepSize() on whichever Integrator has been set as current.

        |  |  |
        | --- | --- |
        | Returns: | the step size, measured in ps |
        | Return type: | double |

    `setStepSize`(*self*, *size*)¶
    :   Set the size of each time step, in picoseconds. This method calls setStepSize() on whichever Integrator has been set as current.

        |  |  |
        | --- | --- |
        | Parameters: | **size** (*double*) – the step size, measured in ps |

    `getConstraintTolerance`(*self*) → double¶
    :   Get the distance tolerance within which constraints are maintained, as a fraction of the constrained distance. This method calls getConstraintTolerance() on whichever Integrator has been set as current.

    `setConstraintTolerance`(*self*, *tol*)¶
    :   Set the distance tolerance within which constraints are maintained, as a fraction of the constrained distance. This method calls setConstraintTolerance() on whichever Integrator has been set as current.

    `step`(*self*, *steps*)¶
    :   Advance a simulation through time by taking a series of time steps. This method calls step() on whichever Integrator has been set as current.

        |  |  |
        | --- | --- |
        | Parameters: | **steps** (*int*) – the number of time steps to take |

    `__delattr__`¶
    :   x.\_\_delattr\_\_(‘name’) <==> del x.name

    `__format__`()¶
    :   default object formatter

    `__getattribute__`¶
    :   x.\_\_getattribute\_\_(‘name’) <==> x.name

    `__hash__`¶

    `__reduce__`()¶
    :   helper for pickle

    `__reduce_ex__`()¶
    :   helper for pickle

    `__sizeof__`() → int¶
    :   size of object in memory, in bytes

    `__str__`¶

High performance molecular simulation on GPUs


### Navigation

- Application Layer
- Library Layer
  - Core Objects
  - Forces
  - Integrators
  - Extras

©2015, Stanford University and the Authors.
|
Powered by Sphinx 1.3.1
& Alabaster 0.7.7
|
Page source
